# Medical student perceptions of establishing effective clinical communication: a qualitative study

**DOI:** 10.1007/s10459-025-10468-x

**Published:** 2025-08-14

**Authors:** Kathryn Veazey, Andrew Notebaert, Ellen M. Robertson

**Affiliations:** 1 Department of Neurobiology and Anatomy, Drexel University College of Medicine, 50 Innovation Way, Wyomissing, PA 19610 USA; 2 College of Health and Human Sciences, Northern Illinois University, DeKalb, IL USA; 3 Department of Physician Assistant Studies, Randolph-Macon College, Ashland, VA USA

**Keywords:** Medical education, Minority stress, Intersectionality, Identity concordance

## Abstract

High-quality delivery of healthcare requires effective communication between patients and providers. Clinical communication has been a priority in medical education for decades, yet studies continue to show that trainees and providers struggle to communicate effectively in real and simulated clinical scenarios. Many have investigated potential methods to improve the initial acquisition, long-term retention, and holistic evaluation of clinical communication competency in medical trainees. However, there has been a lack of rigorous qualitative research investigating the medical student perspective on this process. This study aimed to identify medical student perceptions of what factors may influence effective clinical communication and how those factors evolve with time. Twenty-two medical students were interviewed between August 2021 and May 2022 at the University of Mississippi Medical Center. Their interviews were transcribed and analyzed using deductive, inductive, and categorical coding methods to construct themes. These themes identified six influencing factors (personal identity; biases and assumptions; norms and expectations; language; episodic contexts; and comfort and trust) and one impact (evolution of personal identity). The relationships between these themes enabled the researchers to establish a preliminary model that demonstrates how medical students believe effective clinical communication occurs. Medical educators may use this model to develop innovative curricular objectives, assessment methods, and teaching strategies that might improve student’s acquisition and demonstration of effective clinical communication. These improvements may be most beneficial for students who face minority stress, as well as those combating oppressive systems based on the complex relationships between their multiple minority identities.

**Supplementary Information:**

The online version contains supplementary material available at 10.1007/s10459-025-10468-x.

## Introduction

Effective doctor-patient communication is a necessary skill to provide quality patient care (Graf et al., 2017; Lim et al., 2013; Simpson et al., 1991; Zangeneh et al., 2021). Successful communication encourages patients to share their experiences with their healthcare team, resulting in improved health outcomes, increased patient satisfaction, and decreased disparities (Lee et al., 2009; McKenzie, 2016). Despite the role of communication for clinical practice, many practitioners and students demonstrate clinical communication deficiencies that neither they nor their supervisors recognize (Boulet et al., 2002; Loureiro et al., 2015; Simpson et al., 1991). These discrepancies are fueled by multiple barriers to communication education in healthcare, including a lack of formal training experiences during clerkships, emphasizing content over process, practice gaps between models and reality, and inexpert teachers (Kurtz et al., 2005; Rosenbaum, 2017).

The teaching and assessment of clinical communication skills were dramatically shaped by the publication of the 1991 Toronto Consensus Statement and the 2004 Kalamazoo II Report (Duffy et al., 2004; Simpson et al., 1991; Skelton, 2005). These reports identified essential communication skills, provided evaluation checklists, and identified additional assessment strategies such as direct observation, simulated encounter rating, and patient questionnaires (Duffy et al., 2004; Skelton, 2005). More recently, the 2024 Glasgow Consensus Statement builds upon the foundation set by the Kalamazoo Report while tailoring its model to apply to all healthcare providers who have clinical encounters with patients (Makoul et al., 2024). It also emphasizes the need to treat patients as whole people, instead of working to simply ‘fix the parts’ (Makoul et al., 2024). This model encourages the use of the Communication Assessment Tool, which was published by Makoul and colleagues in 2007 (Makoul et al., 2007). This checklist-based tool can be completed by the patient and physician for self-assessment and has been used across many countries with many clinical provider populations (Makoul et al., 2024).

For medical education in the United States, the advent of the Step 2 Clinical Skills Exam spurred evaluation checklists to rapidly become the dominant assessment method, leading many to question the appropriateness of teaching clinical communication as discrete skills. Some argued that this approach was too reductionist, omitted vital communication behaviors, and could result in inauthentic communication and a mockery of caring attitudes (Mendick et al., 2015; Perron et al., 2015). Due to the recent discontinuation of the Clinical Skills Exam, some have suggested that the time is ripe for transitioning away from checklists and overhauling the way that clinical communication is taught and assessed in undergraduate medical education (Howley & Engle, 2021).

Several studies have investigated discrete factors that may influence the acquisition of clinical communication skills in undergraduate medical education. Some studies have investigated the impact of age or year in the medical program, but often produce contradictory findings (Lim et al., 2013; Lumma-Sellenthin, 2012; Perron et al., 2015; Rees & Sheard, 2002; Taveira-Gomes et al., 2016). Racial and ethnic identities have been the subject of abundant discussion (Lee et al., 2009; Leyerzapf et al., 2015; Osborne, 2001; Rees & Sheard, 2002, 2003; Teherani et al., 2018). Studying gender differences has been a hallmark for measuring communication for decades, with many suggesting that women communicate more effectively than men (Graf et al., 2017; Lumma-Sellenthin, 2012; Rees & Sheard, 2002; Taveira-Gomes et al., 2016). Over the last decade, sexual orientation has become a focus of communication research, with several advocating for LGBTQIA + persons to be included in underrepresented in medicine categories and being subject to minority stress (Eliason et al., 2011; Sánchez et al., 2015; Sitkin & Pachankis, 2016). Additional factors may include experiences with illness or disability (Stergiopoulos et al., 2018), religiosity (Curlin et al., 2006; Woods & Hensel, 2018), being an immigrant or child or immigrants (Lee et al., 2009; Leyerzapf et al., 2015), having doctor-parents (Rees & Sheard, 2002), being a first-generation medical student (Brosnan et al., 2016), and personality (Lievens et al., 2002).

The existing body of work into clinical communication also features several prominent theories. Minority stress theory suggests that additional stressors are placed on those with marginalized identities, including gender, race or ethnicity, and sexual orientation (Cyrus, 2017; Sitkin & Pachankis, 2016). Identity concordance describes when a patient and their provider share one or more characteristics, resulting in feelings of similarity (Moore et al., 2023; Street et al., 2008). Within this theory, there are several subdomains, such as racial concordance, gender concordance, language concordance, and social concordance, each of which has been explored in terms of physician-patient communication. Social concordance describes the impact of similarities across multiple shared identities, such as race, gender, age, and education (Thornton et al., 2011). The relationships between multiple shared identities and their influence on enabling new forms of social exclusion can be described by intersectionality theory (Leyerzapf et al., 2015). This theory focuses on the unique types of oppression and discrimination that are experienced by persons with multiple marginalized identities, such as LGBTQIA + women of color (Cyrus, 2017). Each of these theories relates to identity and lived experience, which may influence how medical students define and demonstrate clinical communication.

To study how medical students communicate in clinical settings, the authors elected to identify a framework for general communication to guide the research process. The Transaction Model of Communication (TMC) was developed in 1970 and is widely accepted as the modern model of communication (Barnlund, 1970; Lapum et al., 2020). The TMC states that communication is the process by which communicators generate social realities and co-create meaning (Barnlund, 1970). Communication is bidirectional, dynamic, continuous, interdependent, unrepeatable, irreversible, and adapted to contexts (Barnlund, 1970). These contexts include social contexts (ex. Rules and norms), relational contexts (i.e., Interpersonal history between communicators), and cultural contexts (ex. Gender, race, class).

This study aimed to identify medical student perceptions of the factors that influence effective clinical communication and how those factors evolve over the course of the medical school experience. To do so, the following research questions were developed:

1. What factors influence how medical students communicate in clinical contexts?

2. How does the medical school experience influence how those factors evolve?

## Materials and methods

### Data collection

The study methodology was purely qualitative using phenomenology. This approach was grounded in an interpretivist paradigm which utilized relativist ontology and subjective epistemology (Alharahsheh, 2020). The researchers decided to base their study within interpretive phenomenology at the earliest stages of conception. They felt this approach would enable them to gain valuable insights into the subjective medical student experience of undergoing clinical communication training and developing as clinical communicators. The authors engaged in a relativist ontology, wherein there is no singular truth but instead realities may be created by multiple intangible mental constructions (Moon & Blackman, 2014). Additionally, they utilized a subjectivist epistemology, which states that meaning is created by the subject imposing meaning on an object or experience (Moon & Blackman, 2014). KV identifies with relativism and subjectivism. EMR identifies with the constructivist paradigm, with critical realism ontology and subjectivist epistemology. AJN identifies with relativism and subjectivism. The authors conferred during the analysis and manuscript preparation process regarding the interplay between their personal theoretical stances and the selected research methodology.

Interpretive phenomenology seeks to describe the subjective experiences that people encounter and create meaning from (Nayar et al., 2014). The phenomenon of interest in this study was to discern how medical students perceived the relationship between effective clinical communication and their own subjective experiences as medical students. The first author (KV) conducted semi-structured interviews with first- through fourth-year medical students from August 2021 to May 2022. Interviews were guided by a script created by KV and two senior educational researchers (Supplement 1). This script was informed by the Transactional Model of Communication, as well as a literature review on clinical communication skills in international healthcare students. These resources suggested that communication is shaped by six factors: physical, physiological, psychological, social, relational, and cultural (Barnlund, 1970; Cargill, 2019; Lapum et al., 2020). The descriptions of these factors were evaluated, deconstructed, and reformatted for delivery in an interview script framework. The script was piloted with two first-year medical students and one healthcare professional, after which it was revised for clarity and flow (Veazey, 2023).

Students were recruited from the University of Mississippi Medical Center (UMMC) via convenience and snowball sampling methods. All first- through fourth-year students were emailed a description of the study, consent documents, and an optional demographic questionnaire. Completion of the questionnaire indicated consent to participate. Participants who completed the consent form were contacted by KV to schedule an interview. Following the interview, participants were asked to forward the study’s recruitment letter to two other students.

Interviews were conducted in-person in KV’s private office, via Microsoft Teams, or via Google Voice. For all but one interview, no other individuals were present.^1^ Each interview began with introducing research personnel and providing a study overview. All interviews were completed in one sitting. Participants were given the option to ask any clarifying questions about the researchers and the study itself. This study was approved by UMMC’s Institutional Review Board (IRB_2021V0546).

### Data analysis

Data were collected until the researchers determined that data saturation had been reached. Data saturation refers to when no new significant findings emerge from the data gathering and analysis process (Fusch & Ness, 2015). All interviews were conducted, recorded, and transcribed by KV. KV took field notes during the interviews, which were referenced during the transcription process. Transcripts were sent back to participants within 3 months of completing their interview for member checking. Participants were offered the opportunity to review their transcripts for accuracy and informed that failure to do so would result in the analysis of their transcript as-is.

The privacy of participants was protected through various means. Consent documents and demographic information were solicited and stored on REDCap, which was only accessible by EMR and KV. Communications about scheduling and member checking occurred through secure institutional email. Identifying information was removed from transcripts immediately following transcription. All names and email addresses were removed from the remaining data three months after the final interview. All published information has been de-identified (Veazey, 2023).

The transcripts underwent thematic analysis according to the steps outlined by Kiger and Varpio (2020). This study used four phases of coding. The first phase involved the first and third authors (KV and EMR) deductively coding the first ten third- and fourth-year transcripts in chronological order then comparing their interpretations for consistency. The deductive codebook was built based on the TMC, which has been used previously to investigate communication in healthcare fields (Cargill, 2019; Paige et al., 2018). Then, clean copies of all third- and fourth-year transcripts were inductively coded using In Vivo and Concept Coding (Saldaña, 2016). The third phase of coding involved the consolidation of all existing codes into categories. This study defined codes as the most basic elements of raw data that can be meaningfully assessed to learn about the phenomenon of interest. Categories were collections of related codes that implied some shared meaning or experience. These categories were then used to construct themes, which explicitly defined the relationships between categories that captured the essence of the data. KV conducted rounds two through four of the coding process, while EMR coded a subset of transcripts to check the codebooks for consistency (Veazey, 2023).

The transcripts of the first- and second-year students were coded using the categories, with new categories being introduced and old categories redefined or eliminated as necessitated by the data. The final round of coding involved using the expanded categories from the third round to re-code the transcripts from the third- and fourth-year students. This final phase was intended to ensure consistency in applying the codes, as well as provide the investigators with a more robust understanding of the results. The coding process was managed through Dedoose version 9.0.62, a cloud application for managing, analyzing, and presenting qualitative and mixed-method research data (SocioCultural Research Consultants, 2022). All authors were involved in the construction of themes. Participants were not asked to provide feedback on the finalized themes (Veazey, 2023). This study utilized the COREQ checklist as an initial assessment of the thoroughness of this report (Tong et al., 2007). This checklist can be viewed in Supplement 2.

The team presents the following as additional strategies to ensure trustworthiness in qualitative research (Ahmed, 2024) (Table 1):

**Table 1:** Methods to Ensure Trustworthiness

Trustworthiness Component	Stegy used by Researchersrat	Detailed Information
Credibility	Prolonged engagement	The authors built trust and rapport with all participants through previous relationships with study investigators. Relationships included peer (KV), teaching assistant (KV, EMR), and faculty instructor (EMR, AJN). The interviewer (KV) additionally built rapport throughout the duration of the interview, with many participants expressing gratitude at the end of their session for offering a safe space to express their experiences.
	Reflexivity	Following expert recommendations, the authors have reflected on their position relative to the research to account for how their subjective interpretations shaped their inquiry and analysis (Olmos-Vega et al., 2023). The full reflexivity statement can be viewed in Supplement 3.
	Triangulation	The authors employed various means of data collection, including interviews, field notes, memos, and frequent reflective discussion. All notes and files were analyzed throughout the thematic process.
Transferability	Thick descriptions	The authors have attempted to provide rich descriptions of the research context, participants, methods, and themes to allow readers to form their own interpretations of the applicability and relevance of this study to their circumstances.
	Sampling strategies	A detailed description of the sampling procedures has been outlined in this study. Additional details can be found in the dissertation format of this manuscript (Veazey, 2023).
Dependability	Methodological documentation	The research team thoroughly documented each step of the research process, including the rationales behind these steps. These documents were consistently updated and referenced throughout the thematic process.
	Audit trails	A record for methodological changes, codebook changes, and thematic changes was kept throughout the process to allow for critical reflection, provide insights into researcher’s positional biases, and ensure dependability.
Confirmability	Peer debriefing	KV frequently consulted with the additional authors, as well as peers from her graduate program and other medical school faculty, to validate the interpretations of the study. The findings of this study were presented in a public format in spring 2023, where a variety of peers and professionals provided additional feedback on the findings.
	Member checking	Participants were contacted following transcription of their interviews to ensure their experiences were appropriately represented. They were also given the opportunity to correct and otherwise alter their transcripts.
	Reflexive journaling	KV kept extensive memos throughout the research process to allow for constant comparison between the researchers’ initial perceptions, interpretations of the data, and theoretical positioning relative to the research project. These memos were reviewed throughout the construction and revision of codes and themes.

## Results

Participants were recruited from UMMC, which is the only academic medical center and allopathic medical school in the state. Its geographic location and chronically health-impaired population often lead to UMMC being the sole provider of advanced healthcare to Mississippians. UMMC matriculates approximately 165 students into its first-year medical cohort each year.

At the time of this study, the medical curriculum at UMMC was divided into a two-year preclinical phase followed by a two-year clinical phase. The first phase included foundational sciences and clinical skills coursework. This phase was dominated by lecture-based teaching through a series of isolated courses (ex. Gross anatomy, pathology, disease prevention, etc.). To begin learning clinical skills, students took Introduction to the Medical Profession (IMP) and Medical Neuroscience and Behavior (MNB). The MNB course incorporated case-based learning, while the IMP course introduced history-taking, medical interview skills, rapport-building, note-taking, and professional identity development (Veazey, 2023). Students were evaluated through a series of standardized patient encounters, as well as by faculty preceptors who offered them supervised guidance during patient visits towards the end of their second year. The MNB and IMP courses also incorporated a set of simulation experiences to broaden students’ experiences, including topics such as poverty, substance abuse, and interprofessional communication. At the end of the preclinical phase, students were expected to pass the United States Medical Licensing Examination (USMLE) Step 1.

During the clinical phase, students rotated through different departments at university-affiliated hospitals and clinics to learn via direct patient care (UMMC, 2022). During their third year, they rotated through six core clerkships, each of which featured two Objective Structured Clinical Examinations (OSCEs). OSCEs consisted of timed standardized patient encounters, followed by writing a patient note. Students were evaluated by examiners using a checklist rubric, with some clinical sites also incorporating a global rating scale. Global rating scales aim to offer a holistic evaluation of student performance without using a checklist. In their fourth year, they rotated through eight specialty clerkships, of which four were required. At the end of the clinical phase, students were expected to pass the USMLE Step 2 Clinical Knowledge Exam. Prior to 2021, there was an additional licensing exam – USMLE Step 2 Clinical Skills (CS). The Step 2 CS exam formerly consisted of twelve OSCEs across the span of eight hours. It was discontinued as a result of the Covid-19 pandemic, but had made a lasting impact on the way that clinical skills have been taught and assessed in American medical schools.

### Description of the sample

Twenty-two interviews were conducted. These consisted of six M4s, four M3s, six M2s, and six M1s. Interviews lasted between 41 and 155 min (average = 76 min). Seven participants verified the accuracy of their transcripts, with two making minor revisions. No participants dropped out of the study following their interview. Participant ages ranged from 23 to 39 (average = 26 years). They came from diverse sociodemographic backgrounds, with the majority identifying as White or Caucasian, heterosexual, and female. Altogether, participants identified as the following: cisgender male, female, male; African American, Asian, Black, Caucasian, Kenyan American, North African Middle Eastern, White; bisexual, gay, heterosexual, pansexual, queer, and straight. Most participants also identified as first-generation medical students and as religious.

### Results of thematic analysis

The initial deductive codebook contained 100 codes to be applied to the third- and fourth-year transcripts. The inductive phase of coding produced 1038 additional codes, resulting in 1138 total codes. These codes were consolidated into 67 categories. These categories were used to construct six themes which identified factors that medical students believed to influence effective clinical communication, as well as one theme describing the impact of the medical school experience. The themes that identified influencing factors were named as follows: (1) Personal Identity; (2) Assumptions and Biases; (3) Expectations and Norms; (4) Language; (5) Episodic Contexts; and (6) Comfort and Trust. Theme 1 was divided into three subthemes: 1a) Extrinsic Identity; 1b) Previous Lived Experiences; and 1c) Intrinsic Identity. One theme was constructed for the impact of the medical school experience: (7) Evolution of Personal Identity. This theme had four sub-themes: 7a) Influence on Intrinsic Identity; 7b) Impact on Mental Health; 7c) Building Awareness; and 7d) Developing Clinical Preparedness. Themes have been presented as a description followed by supportive quotes from participants.

#### Theme 1: personal identity

This theme was defined as the external and internal characteristics of an individual which, combined with the weight of their lived experiences, shape their perceptions of and interactions with their world. These factors generally were grouped into three sub-themes.

*Extrinsic Identity* described the identities that have been traditionally associated with external characteristics and behaviors. These identities include gender, age, race, ethnicity, and sexual orientation. It is worth noting that many of these identities are not readily apparent to others or may appear different to outsiders than how a person perceives them. This theme defines how a person’s knowledge of their own extrinsic identities (i.e., ‘Who I look like to the world”) shapes the way that they approach interactions with others; it is not about how others react to a person’s external identity.

Being gay, I feel like I know what it’s like to have every aspect of your communication scrutinized and put under a microscope. So I want others to feel not that when they communicate. I know what it’s like to be dismissed, so I want to make sure others don’t feel dismissed. I know what it’s like to not be heard, so I want other people to feel heard. – M1-3.

Concordant extrinsic identities were often cited as allowing students to build rapport and empathize more readily. When experiencing discordant extrinsic identities, participants frequently noted that this led to an increased reliance on other factors, such as leaning into assumptions or meeting someone’s expectations.

I tend to be a little bit more cognizant of people’s identities than sometimes others are, especially when it comes to race … If there is a person of color and they’re like looking to me in a room of like people who don’t look like us, being like “oh what does like that girl think?” – M4-2 (identifies as Asian).

*Previous Lived Experiences* encapsulated the choices that individuals made and how those choices shaped their perception of themselves and their world. Frequently cited experiences included being a parent, married, a first-generation medical student, an immigrant or child of immigrants, and experiencing personal illness or tragedy. These experiences changed how participants thought about themselves, their communities, and others around them. For example, participants who lost a loved one to disease or suffered personally from chronic pain or acute illness shared how those experiences allowed them to form deeper, more meaningful relationships with patients experiencing their own losses.

I was like hospitalized for sepsis at one point. Um, so I know like how I would have wanted to feel when I left, ‘cause like I didn’t know what had happened while I was in the hospital … So I feel like I look at it from that perspective of like, “What would I want to know if I’m in their shoes? How would I want to feel leaving this encounter? What questions might I have if I were them?” And then kind of get all those answers together before I go in to talk to them. – M3-2.

While the experiences were discrete, their impacts were more nuanced, such as increasing awareness of privilege, building communities of support, and acknowledging one’s own strengths and limitations.

I grew up in a household where … my dad did not speak English as a, as a first language. … Me and my brother … were speaking in a different language all the time … I do identify a lot with immigrants, I think because my dad was one … so when it comes to um [non-English speaking patients], I feel like you have a responsibility to them.” – M2-3.

Prior to having a son, I don’t think I like communicated well during like pediatric rotations … It was harder for me to not only gather history from like a child, but like knowing how to talk to parents specifically about a child. And I think me now as a mother, that has made all the difference. – M4-6.

*Intrinsic Identity* referred to the abstract, unquantifiable concepts that resulted from the culmination of experiences and interactions a person had engaged with. These concepts included empathy, humility, personality, sociability, attitudes towards learning and communication, and reasons for pursuing careers in medicine.

My main reason [for pursuing medicine] is to be able to help and serve people, and so if that’s my main reason, then like communicating with them is the best way for me to actually help them. – M1-2.

If you have someone coming in, they’re asking a lot of questions, they know a little bit about their situation, it’s like a little bit easier to kind of pinpoint what they know and what they don’t know. Like if you have someone who comes in and who’s like kind of tight-lipped … Totally different personalities, you know? So. But that’s the part that really makes a difference. – M3-4.

If I [have] good attitude about being taught clinical skills or like clinical communication skills then I’m more likely to use what I learned to talk to patients … Thinking of it as a skill makes it something that you improve on. Hopefully. – M4-1.

These factors shaped the way that participants thought about themselves, their experiences, their values, and their goals. For example, many participants emphasized the need for humility and adaptability to have formative conversations – a person must be willing to exchange ideas with another, acknowledge the inaccuracies in one’s own previous perceptions, and adjust their framework to accommodate a new position. Stubbornness and pride were often cited as examples of poor communication types.

I value humility a lot and always believing that you have something to learn. And so uh if you’re humble, you can always revise your views. You can always learn something. You can always grow … If you believe you’ve learned everything there is to know, you’re stagnant. There’s no more growth, you’re not learning anything else, you’re not expanding your abilities. – M2-1.

#### Theme 2: assumptions and biases

The previous theme described a given individual’s determination of their identity; this theme described how others determined a person’s identity (i.e., “How the world thinks I look”). Assumptions referred to internal processes about another person that were assumed to be true in the absence of supporting evidence. Biases referred to when those assumptions resulted in changes in communication behaviors. These changes could be positive, negative, neutral, intentional, unintentional, conscious, or unconscious.

So I’m Black and sometimes … if it is my patient… like their responses are either not going to necessarily be directed towards me or um when I’m coming in to get the preliminary information um, they will be kind of obviously shocked or entertained that I am part of their medical team. – M3-3.

Assumptions and biases were described as occurring bidirectionally, being often unavoidable, and always worth mitigating. As one participant stated, assumptions damage the connection between providers and their patients:

Assuming like maybe your patient’s sexuality or their um substance use history or anything, like making assumptions about people. Any assumptions … can kind of like fuel the disconnect with the provider and the patient. – M1-6.

Some examples of factors that people made assumptions and biases about included gender, race or ethnicity, religious affiliation, height, age, and socioeconomic class. The latter examples were cited as being often overlooked experiences that many students faced, such as the assumptions made about tall, bearded men and the appropriateness of them working in gynecological offices. The weight carried by religiosity and class were strongly felt by participants and often situated in the specific social context of being in the Southern United States.

I have such like strong views about Christianity like having been brought, brought up in a Catholic family and just like some of the like negative feelings that I’ve been having lately towards it, like that’s something that I need to work on because I don’t need to have biases towards people who are Christian. – M1-4.

A lot of people in our class are from families with more money … If you and your patient both are in the same bracket or whatever, whether they be Black or White, I think it’s easier to communicate, so I think that that, that shouldn’t be a problem for most of us, communicating with people of our own socioeconomic status. – M2-4.

#### Theme 3: expectations and norms

External to the individual communicators were broader societal and cultural expectations and norms. Expectations described one’s feelings about how something should happen, typically based on history of past behaviors or events. Norms entailed social behaviors that are expected of a person or group of people based on their presence as part of a specific group or culture (for example, participation as a medical trainee at an emergency medicine clinic).

When you first uh, interact with patient, um, set some expectations so that their emotional state is not compromised … Especially if they were in a really bad mood and had to wait in the ER a really long time and don’t know what’s going on … Starting those conversations about setting expectations to further facilitate fully the exchange of information effectively. – M4-4.

Expectations and norms were shaped by dominant cultural values, often of a department, institution, or clinical location. These factors could be influenced by the roles that people were expected to assume in different situations, as well as the prevalence of protocols and scripts to abide by in clinical and educational settings.

I would say part of the errors in communication I think were built from not um, stating any expectations whatsoever on that part of the rotation, so I will say it’s easier to communicate when your role has been like clearly defined. And you know, because residents change so much, you know exactly what they want from you and like how they want you to approach things. – M3-2.

Examples include knowing when to speak up versus when to listen, spending adequate but not excessive time with patients, and determining when to use honorifics or ask for pronouns. Abiding by explicit and implicit norms facilitated rapport, diffused antagonistic situations, and allowed for efficient delivery of care, especially in large care team settings.

I think you are always taught how you be polite in conversations like the “Yes, ma’am, yes, sir. No ma’am, no, sir.” Which I’ll also have to really remember, like not everybody wants to be called, “Yes, ma’am” … so like I’ve learned … how to read the room and know when somebody’s going to be expecting that from me. It kind of like definitely like changes like the way I talk to people way older than me. Or just a little bit older than me. – M2-6.

#### Theme 4: language

The theme *Language* encapsulates the verbal, nonverbal, and written behaviors that people used to establish communication. This theme included English and non-English languages, body language, and professional versus layperson’s language. It also involved the means of transmitting language between communicators, which included face-to-face, telephone, and video calls.

I can’t imagine like you know, being sick and wanting to get healthcare and not being able to speak like the same language. It’s gotta be like so scary, so frustrating. But there was one time whenever the translation service like was not working and … we actually had to call in someone who was fluent um in Spanish … With Spanish like I can introduce myself and you know, like I can form simple sentences, but I’m in no way able to like actually communicate … Same with ASL. – M1-1.

Whatever your job is, you have jargon that’s specific to it. So if you’re using terminology that that person doesn’t understand um, I would think that would be an example of, of bad communication. –M2-5.

Several participants described the benefits and challenges of being explicitly taught non-verbal communication skills, such as appropriate levels of eye contact, limb positioning, use of gestures, and how to orient one’s face and torso to convey active listening.

I didn’t realize there was a - not a one-size-fits-all, but a very standardized way to present with body language for the patient to generally feel most comfortable … I always thought, you know, body language just kind of, just kind of happened … It’s legs not crossed. Don’t cross your arms. Uh, don’t have anything between your face and the patient’s … Don’t be all in their space but at the same time, don’t be all laid back, relaxed and there’s just a lot of little things like that. – M1-5.

Participants also mentioned the impact of the Covid-19 pandemic on their language, specifically with reference to how it changed their nonverbal behaviors due to mask usage, as well as their increased need to gain proficiency with telehealth communication.

It was like the summer of 2020, so it, it was a very unique like shifting time … And just being able to communicate with masks when like we were just starting to wear them all the time … makes it more important like to have the facial responses in your like upper face. But it can also make it difficult for people who don’t hear as well, um. And I think that even if you like technically don’t have a hearing impairment, we were relying on like the whole face, um. And so it’s kind of changed how we communicate. Probably more people use gestures than before. – M3-3.

#### Theme 5: episodic contexts

The people involved, their location, the topic of communication, and the mood of the participants all influenced how effectively the communication occurred. These were grouped as *Episodic Contexts*, which referred to the transient factors that varied between each communication encounter.

When you go in and speak with a patient, you want to be somewhat personable. And like not that you’re not with your clinical care team, but it’s like time is so strapped. But like at the same time, like with your patients, you do, I do think that you have um like the obligation to make some personal time with them, especially because I do think that’s kind of critical to establishing rapport… Whereas I feel like in a clinical team, we can all kind of share a sense of urgency together, so it can, if it needs to be like short and to the point and like you know, it will be. – M2-3.

Additional episodic contexts included whether explicit time constraints were present, whether there was a prior existing relationship between communicators, and the physiological state of communicators. Sickness, stress, fatigue, hunger, and thirst were referenced as limiting factors in establishing effective communication, regardless of which communicators experienced them.

A lot of times, people in the hospital, they’re sick. They may or may not act like they normally would. They may be confused. They may not be very kind because they don’t feel like it, and that’s OK You get people who may not be very kind to you. Maybe not because they’re sick, but just because they’re not being very kind that day, uh. And you know, you, you learn how to deal with that, and I think that’s a good skill to have too. – M2-5.

Within the realm of clinical communication education, students also referenced the authenticity of the encounter as heavily impacting their communication. Many students specifically mentioned OSCEs and standardized patient encounters as being inauthentic, which negatively impacted how effectively and efficiently they were able to communicate.

While the like standardized patient interviews do help… sometimes it can be kind of annoying … I still think that the actual clinical experience in the third year … really is where it comes together … There’s no substitute for just actually talking to a real patient and a real person … You can’t simulate that. – M4-3.

#### Theme 6: comfort and trust

Students defined effective clinical communication in terms of their ability to establish rapport with patients, patient’s family, and other healthcare providers, which then enabled mutual decision-making aimed at improving their patient’s health. Participants emphatically stated that effective rapport-building could not occur without establishing an environment of comfort and trust. *Comfort* described feelings of ease and relaxation or the alleviation of distress. *Trust* described a person’s belief in someone else’s willingness and ability to help them in moments of distress.

I have definitely learned in the hospital to be more comfortable … Everyone creates a comfortable environment for the patient, like ensuring that that’s happening … ‘Cause even if I do my best to make … the patient feel comfortable, it only takes like one person to like make them feel uncomfortable. – M4-2.

Participants repeatedly cited that good doctors were able to convince others to trust them and subsequently, their judgement. In the absence of comfort or trust, effective communication broke down, which hindered the provider’s ability to provide patient-centered care.

I’ve seen things that are either very awkward and uncomfortable, trying to talk about sexual histories and drug use histories and things like that, that the person asking the question was uncomfortable, the patient was uncomfortable, and I feel like the information was not effectively shared and gathered in that encounter because of the amount of discomfort. – M4-5.

To create comfortable and trusting environments, participants recommended demonstrating compassion, cultural sensitivity, empathy, humility and adaptability, forgiveness, honesty, patience, and professionalism. Participants spoke of these traits as skills to be trained, as opposed to innately present. The continuous effort to master these behaviors was, as many participants put it, the key difference between a “good” doctor and a “great” doctor.

Empathy. If you can’t feel for your patients, you’ll never understand what they’re feeling. And if you can’t understand what they’re feeling, you’ll never understand why they’re doing what they’re doing, And so if you can’t meet them there, not halfway, but where they are, then you’re just gonna be running on a treadmill with some people. They’re never gonna get better, and they’re never gonna understand - you never understand them and they can’t understand you. So empathy is really, is necessary to be a good doctor. And that’s hard. – M3-1.

The presence of comfort and trust was directly associated with patient outcomes, in that patients listen and learn best from those they feel they can connect with. The ability to connect with patients spanned across multiple specialties and care settings, but was equally impactful for all participants at some point in their training.

I feel like most of that is just what service I’m on … I don’t put as much like, uh effort into [some services] as I do on services like house medicine, where you’re the primary team and you’re going to see them every morning and you’re going to be the one who communicates bad news to them. You’re going to be the one who like, basically, like establishes enough trust that they stay in the hospital … and when they leave they keep doing like what they need to do to get better. – M4-1.

#### Theme 7: evolution of personal identity

This theme captured the relationship between progressing through UMMC’s medical training and the medical student themselves. The medical school experience altered the identities of participants, especially along four specific components: intrinsic identity, mental health, awareness, and feelings of clinical preparedness.

Given the previous definition of intrinsic identity as abstract concepts that included empathy, humility, and personality, the sub-theme *Influence on Intrinsic Identity* focused on the ways that many, if not all, of these factors changed with continued exposure to the field of medicine. Many students described that their empathy and humility had drastically improved with clinical exposure, especially when working with historically marginalized and disadvantaged communities. Many spoke of how their personalities had shifted more towards extraversion as a result of the constant need to self-advocate and network. Students also described the acquisition of introspection skills, which allowed them to critically reflect on their communication encounters and work to continually improve their approaches.

I think coming into medical school, I was a lot more timid … I think now um I’ve learned to kind of step up … I think I’m a lot more confident in myself and my abilities um just from training. And yes, I think that for sure has made me more outgoing … I think it kind of forces you to, especially during your fourth year and like residency interviews and the need to network. I think you’re kind of like pushed to like talk and speak up. – M4-6.

*Impact on Mental Health* portrayed the continuing struggle that students faced to maintain their mental well-being in response to the many rigors and stresses of medical school, as well as personal circumstances like mental health diagnosis (ex. ADHD, anxiety, depression). Students often described their mental health changes as negative, but also spoke about improvements in their knowledge of and willingness to use mental health resources, combat mental health stigma, and advocate for recognition of and proper care for mental health disorders in others.

M1 year … it’s like the perfect settings for a mental health disorder … On test day … I was just so tired, I couldn’t even think anymore. Or I was sad and depressed. And I didn’t even really feel like trying, like I gave up all hope … I feel like two things happened during M2. My learning increased, like my ability to learn increased. And then also my mental health like improved because I wasn’t spending all my time either worrying about doing well or not doing well or not having, like being like completely burnt out. – M2-3.

*Building Awareness* contained students’ frequent references towards becoming more informed about a variety of phenomena, including social determinants of health, alternative career paths in medicine, the history of medicine, and the relationship between demographic variables and healthcare discrimination.

One of the things that was really impactful was when we were being taught about the history of race in medicine in Mississippi … Knowing that I’m going to be treating a population that’s gonna probably be majority Black; knowing their experiences with medicine and how things can be negative and how things can be scary, helped me … actually address their concerns and their fears, so. That’s definitely helped me. – M1-2.

Finally, the sub-theme *Developing Clinical Preparedness* illustrated the process of becoming more confident in one’s own healthcare competency. Many students spoke about their feelings of preparedness in both positive and negative terms. They identified several learning experiences as the most impactful, including working with the Jackson Free Clinic (a student-run non-profit healthcare clinic mainly serving disadvantaged populations), the preceptorship experience, and their clinical rotations. Students had mixed comments regarding their training in OSCE or standardized patient scenarios, with many referencing the poor authenticity and time constraints as limiting their ability to learn and practice effective communication skills. Finally, feelings of clinical preparedness culminated in the formation of a new identity in several students: that of a “future doctor” (M2-2).

My 3rd and 4th year, we see such an incredibly diverse patient population in terms of pathology and also ethnicity and backgrounds and things like that, as well as like educational backgrounds. So I think that I’ve seen just about everything um in terms of like communications scenarios and stuff, so I feel very prepared moving into residency. – M4-5.

## Discussion

Competent healthcare professionals must be able to communicate effectively and efficiently with their patients, patient’s family, and other healthcare providers. This study suggests that identity may serve a central role in establishing environments of comfort and trust, which was described as being essential to the performance of effective clinical communication. Feelings of similarity (concordance), experiences with power systems that perpetuate discrimination and marginalization (minority stress), and the relationships between multiple identities (intersectionality) combine to determine whether communication proceeds transactionally, whether rapport is established, and whether mutual understandings and improved health outcomes occur. In the upcoming section, the authors make several specific recommendations for centralizing identity, the factors that influence it, and its subsequent influence on learning and demonstrating effective clinical communication:

A. Early exposure to explicit language barrier training, including English to non-English and professional to layperson’s language.

B. Expanding simulation to include patients, patient’s family, and other healthcare providers.

C. Evaluate clinical communication using global ratings scales with cultural diversity in mind.

D. Early exposure to diverse cultural minority patient and provider populations.

E. Increased opportunities for interprofessional education experiences to emphasize professional identity development, interprofessional identity development, and interprofessional collaboration.

### Factors that influenced clinical communication

Students identified many factors that they perceived to influence effective clinical communication. These factors began with the individual and grew to encapsulate interpersonal, environmental, and sociocultural factors. Each individual factor contributed to the formation of an environment of comfort and trust, upon which a strong foundation for effective communication and long-term rapport could be built. By identifying these factors, the researchers were able to gain insights into the role of the medical student experience in the development of personal and professional identities. Based on these insights, the researchers were able to generate a preliminary theoretical model for establishing effective clinical communication.

The factors that students identified as influencing clinical communication partially align with the Transaction Model of Communication. The theme episodic contexts included factors such as ambient noise (physical context), whether a relationship exists between communicators (relational context), and the mood of communicators (psychological context) (Cargill, 2019; Lapum et al., 2020). The social and cultural contexts of the TMC were reflected in the relationships between biases, assumptions, expectations, and norms. The theme Comfort and Trust emphasized the need to build rapport, which was also seen in the TMC as it highlighted the creation of new communities and relationships between people. However, the TMC places many of the factors of identity as cultural contexts, which this study suggests may be too limiting an interpretation of the complex nature of identity and its impact on communication.

Students argued that establishing effective clinical communication is a process, not a skill. Within medical education, clinical communication has often been described in terms of discrete, observable skills. Students were often taught via checklists of behaviors to perform, then assessed on similar checklists. However, it has been suggested that this format of teaching and assessing students may lead them to only mimic authentic caring behaviors by demonstrating skills that they feel are necessary to impress their evaluators (Clever et al., 2011). To combat this issue, some have recommended that clinical communication be assessed holistically using global rating scales, as well as account for more subjective factors like intuition and the need to deviate from protocol (Mendick et al., 2015; Salmon & Young, 2011; Skelton, 2005). These scales can accommodate the need to change one’s communication based on the communication style and challenges posed by different patients (Bußenius et al., 2022). To teach for this assessment model, several methods have been suggested, including real or simulated experiences, role modeling, and opportunities for reflective practice (Mendick et al., 2015; Skelton, 2005). However, the use of global rating scales may cause additional confusion, especially in novice learners, who likely want more concrete examples of ‘good’ communication. It is also possible that this approach may present additional difficulties for larger medical schools due to time, training, and resources requirements – especially when considering the wide assortment of preceptor evaluators across many clinical sites.

The identities of communicators were referenced as being foundational to each subsequent step of the communication encounter. Students frequently referenced that one of the first occurrences during the transaction would be for each individual to make assumptions based on the extrinsic, intrinsic, and experiential identities of the others involved. Students encountered altered assumptions and biases based on several specific identities, including racial and ethnic identity, LGBTQIA + identity, and gender identity. Both LGBTQIA + and students of color described how their increasing awareness of the history of medicine helped them understand why many historically marginalized patients may resist seeking healthcare. It is worth noting that students who identified as White- or Caucasian also referenced how their increased awareness of the history of medical malpractice for marginalized groups had led them to becoming extra cautious with patients from those groups. The complex interplay between multiple identities, as well as shared experiences across disparate identities, should be at the forefront of each communicator’s mind throughout the transaction.

Per the students’ shared experiences regarding the interplay of identity with assumptions and biases, gender appeared to be a central concern. Nearly all students who identified as women or female mentioned that others often assumed they worked in the field of nursing instead of medicine. They also reported that many patients and supervisors made derogatory comments based on their physical appearance and perceived competency based on their sex or gender. Students who identified as men or male shared their concerns that their height, build, and facial hair might lead others to assume they had paternalistic, domineering, or uncaring attitudes. Given that the latter of these populations is not often studied in terms of gender discrimination, their experiences with gender bias offered novel insights into the provider-patient encounter.

Feelings of belonging to a minority group were described by participants as producing the effects of minority stress. Minority stress describes the additional structural and interpersonal barriers that disadvantaged groups face in comparison to their advantaged peers (Teherani et al., 2018; Wyatt et al., 2020). Traditionally, this term has been applied to racial, ethnic, and gender minority groups, with a recent impetus to include LGBTQIA + groups (Eliason et al., 2011; Sánchez et al., 2015; Sitkin & Pachankis, 2016). However, this study’s first-generation medical students also shared some of these experiences. Many of the students who identified as first-generation medical students also identified as children of immigrants. The overlap between these identities created additional barriers for them, including a lack of preexisting social capital, feelings of imposter syndrome or not belonging, and challenges adapting to several of the norms of medical school (ex. Prioritizing academic success over personal wellbeing).

The impact of experiential identities could also be seen in those students who shared their experiences as patients. Experience as patient spanned across every demographic group in this study, including gender, race or ethnicity, and LGBTQIA+. These experiences were frequently spoken of in terms of enabling students to build a deeper sense of empathy with their patients, as well as gaining understandings of the long-term consequences of poor communication for patient health. Interestingly, advantaged populations (ex. Heterosexual White cisgender students) discussed the lasting impact that their patient experience had on them. It may be that these students’ experiences as patients allowed them to feel an acute sense of powerlessness or vulnerability that they were not accustomed to, especially compared to historically marginalized groups who may experience frequent vulnerability in healthcare settings. It is possible that experiences as patient may offer effective avenues for learning effective clinical communication in healthcare trainees. Some institutions have started incorporating simulations that place the student in the role of terminally-ill patients or patients struggling with poverty, which have been shown to reduce stigma, generate feelings of compassion and empathy, and increase comfort when discussing challenging topics like end-of-life issues (Elzie & Shaia, 2021; Murray et al., 2022). These experiences may be particularly impactful based on their ability to allow students to acquire a new experiential identity as patient, even if in a simulated environment.

Regardless of a student’s experiences with minority stress, all participants described the relationship of identity to the formation of comfort and trust. When individuals shared identities, rapport could be effectively and efficiently established. This phenomenon has been referred to as identity concordance. Common examples of identity concordance from the students were gender, race or ethnicity, and LGBTQIA + identity, as well as experiences as a caregiver (most often a parent) or experiences as patient. Language concordance was also frequently referenced in terms of its absence. Numerous participants described the additional communication barriers that are present with non-English speaking patients (ex. Spanish or American Sign Language). Religious concordance may also be worth considering, especially in circumstances of its absence. One of this study’s non-religious participants described their continuing need to address their biases towards religious persons and their concerns about their ability to do so as a future healthcare provider in the American South. The location in the American South, as well as the dominance of religion as a form of cultural capital, may be worth critically evaluating for their impact on the study’s data. Several studies have investigated the role of identity concordance in the context of healthcare provision, but to the researcher’s knowledge, none have described the impact of experiential identity or religious identity on clinical communication (Lor & Martinez, 2020; Moore et al., 2023; Thornton et al., 2011).

**Fig. 1:**
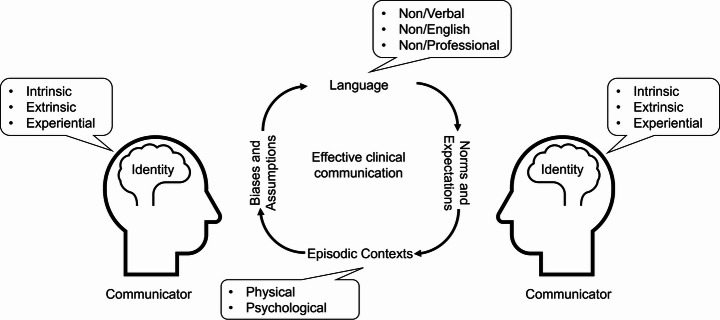
Model for establishing effective clinical communication

The presence or absence of concordance appeared to affect all subsequent phases of the communication transaction. Based on feelings of similarity or difference, students made assumptions about the other person, while that person was presumed to be making their own assumptions about the student. Oftentimes, these assumptions and biases were based on extrinsic identities, which subsequently led to larger assumptions about cultural characteristics like class, educational level, and competency. Each person’s assumptions and biases went on to influence their expectations of one another, including the unstated norms they sought to adhere to and the language that they used to communicate. These norms and expectations were also strongly guided by the episodic contexts of the encounter, as well as the languages used by each communicator involved. The culmination of these factors then defined whether an environment of comfort and trust could be established. In the presence of comfort and trust, effective transactional communication could occur. In its absence, ineffective communication occurred, often mimicking transmission or interaction models. The relationship between these different factors can be visualized in Fig. 1.

### Impact of the medical school experience

Given the role of personal identity as the foundation of effective clinical communication, it came as no surprise that the medical school experience primarily enacted change on students’ identities. Many of these changes were to students’ intrinsic identities, especially empathy and personality. Most of the intrinsic identities that shifted throughout their medical training were not specific to medical student populations; however, certain identities were, such as identity as future doctor. These identity changes culminated in students gaining the ability to describe effective clinical communication, articulate how different factors influence it, and identify areas for personal growth and development as transactional communicators.

Students described the clinical phase of their training as being particularly impactful on their clinical communication. Several mentioned learning through role models during their preceptorship experience, while others shared their mixed opinions about the utility of simulated patient encounters. Many spoke about their volunteer experiences with the Jackson Free Clinic (JFC), a student-run non-profit clinic serving uninsured patients. The JFC and other volunteer experiences offered students early and frequent opportunities to be exposed to diverse cultural and healthcare populations, allowing students to expand their awareness and approach to holistic care. Focusing on the preceptorship experience, students frequently mentioned learning positive and negative behaviors from engaging with role models. These experiences often encouraged students to determine their own internal gauge for appropriate versus inappropriate behaviors; in this way, it is likely that role modeling may exert its dominant effects through its impact on students’ intrinsic identities and subsequently their engagement with norms and expectations. Lastly, regarding simulated patient scenarios, students spoke of the opportunities for immediate personalized feedback to be vital to their awareness of their own abilities but also spoke of the inauthenticity of the encounters and negative impact of time constraints. It is likely that simulated patient encounters will continue to act as a cornerstone of medical training based on their ability to offer timely feedback, an introduction to the language of medicine, opportunities to practice skills related to the patient interview and physical examination, and prompt thoughtful discussion surrounding the complexities of healthcare (ex. Conversations about death, barriers to accessing healthcare, and insurance limitations).

While students rarely described their changes in identity based on their year in the program, they often described the evolution of their role as members of the healthcare team. Some provided explicit examples of the RIME model (Reporter, Interpreter, Manager, Educator), while others spoke in broader terms about their increased clinical responsibilities, expectations of leadership, and enhanced understanding of the roles of other healthcare professionals and their position relative to them on a multidisciplinary care team. Regarding this last point, it is possible that as students became better equipped to articulate their own professional identities as future physicians, they grew to appreciate how others’ professional identities and expertise could benefit complex healthcare scenarios. A recent review suggested that the professional identity of clinicians may influence their ability to practice collaboratively on interprofessional teams due to differences in personal factors (like values and beliefs), their education and professional experiences, and interprofessional socialization (Wood et al., 2022). While this study explicitly focused on medical students, it may be beneficial to explore the complex relationships between some of the factors identified in those populations compared to other healthcare trainees and practitioners. Given the importance of personal identity in shaping medical student perceptions of effective clinical communication, it is likely that interprofessional identities may also play a key role.

### Implications for medical education

This study offered novel insights into the impact of medical student identities on the perceptions and demonstration of effective clinical communication. The researchers were able to enhance the depth of existing knowledge by providing new considerations regarding minority stress, identity concordance, and intersectionality theories. While the latter has traditionally focused on the impact of multiple minority identities, the complex relationships found in the data suggest that critical reflection on intersecting identities is vital for all future healthcare practitioners, especially those with many cultural majority identities. Many of the participants in this study who identified with underrepresented groups spoke of their experiences as being distinct from that of their peers; they were “outsiders” to the cultural majority of medicine. These experiences were often cited by students as helping them build strong connections with peers and patients who shared those experiences. These results may be benefit from study in other healthcare trainee populations – both within and outside of the US – who may benefit from explicit curricular opportunities to discuss the impacts that marginalization, implicit bias, and structural inequities have on the experiences of themselves and their peers (Khazanchi et al., 2021). By involving cultural majority and minority stakeholders in these conversations, it may be possible to begin addressing and dismantling some of the systems that perpetuate community separation and thereby enhance connection and open communication between and across groups.

To reach this goal, this study’s participants made several recommendations for healthcare educators, emphasizing early patient exposure and increased training on cultural awareness and diversity from individuals representing those populations. Making communication training more explicit has been strongly supported in previous works, as leaving it as an implicit assumption may contribute to the disconnect between theory and practice (Rosenbaum, 2017). Participants also requested explicit telehealth training, increased language barrier training, and increased opportunities to practice delivering bad news. Regarding language barrier training, previous studies recommend that providers who have received language concordance training felt more able to identify patient mistrust, reduced their use of jargon, and improved overall communication quality (Lor & Martinez, 2020). For practicing professional language, medical educators can incorporate existing communication frameworks into cadaveric anatomy labs. A recent study did exactly this by applying the SBAR framework (abbreviated from Situation, Background, Assessment, Recommendation) to practicing handoffs between different dissection teams (Lazarus et al., 2016). In this way, students are still able to practice necessary provider-to-provider communication scenarios in low-stakes environments (Lazarus et al., 2016). For practicing delivering bad news, there are several options to pursue, including reflective activities and explicit practice opportunities for navigating uncertainty (Ledford et al., 2015; Meitar et al., 2009).

Several studies have found medical education and assessments that reduce communication skills into discrete behavioral components to be harmful to long-term learning and demonstration of clinical communication (Mendick et al., 2015; Salmon & Young, 2011; van den Eertwegh et al., 2014). Some have recommended that communication encounters should be evaluated using global rating scales, which offer holistic appraisal instead of evaluating disjointed aspects (Salmon & Young, 2011). Others have supported the use of skill-based education, as those skills become “components of a toolkit to be selected and used appropriately” to achieve the shared goals of the provider and patient (Silverman et al., 2017). Whichever rating scales are used, they should be designed for culturally heterogenous students to avoid inadvertently punishing cultural minority behaviors, like avoiding eye contact or demonstrating reticence (Fernandez et al., 2007; Lee et al., 2009). An alternative to global rating scales is the Communication Assessment Tool, which when used in tandem with the principles outlined in the Glasgow Consensus Statement, may be able to provide assessment of a providers’ ability to deliver humanistic, person-centered care (Makoul et al., 2007, 2024). The previous studies acknowledge that the use of checklists may produce ‘check the box’ mentalities, but the guidance provided as to their use and purpose can reframe the narrative to focus on building supportive, empowering provider-patient partnerships (Makoul et al., 2024).

When designing communication skills curricula, courses themselves should be designed with a diverse student population in mind by introducing cultural and language issues early in the first years of training (Rees & Sheard, 2003). They should also include abundant space for reflective practice from both the patients and providers, as the meaning of communication lies in subjective experience, and thus patients and experts may share opposing views on what makes a given communication encounter valuable (Salmon & Young, 2011). Communication skills educators must be aware of the complexity of clinical and communication scenarios encountered by clinicians to better tailor their curricula and allow their students to use their skills flexibility to suit various contexts (Silverman et al., 2017). Students need to be confronted with challenging communication encounters across all levels of training, whether in real or simulated learning environments, to stimulate lasting changes in their behaviors (van den Eertwegh et al., 2014). These confrontations can involve reflection, reviewing audiovisual recordings and noting poor behaviors, or verbal feedback of poor performance (van den Eertwegh et al., 2014). These communication encounters can also be expanded to include not just patients and providers, but also patient family and other members of the healthcare team. An example of the former would be adult triadic interviews presented by Shibli-Rahhal and Kreiter (2021). Lastly, curricula should emphasize the need to approach communication from both a skills- and attitude-based approach. Skills-based approaches help students learn what behaviors are appropriate and how to rehearse those behaviors. Attitude-based approaches focus on the need for self-awareness and reflection to explore how the doctor’s thoughts, feelings, and emotions towards patients influence their interactions with patients (Kurtz et al., 2005).

Exposure to cultural minority populations may be the key step towards improving clinical communications curricula. These populations include racial and ethnic minority groups, sexual and gender minority groups, and non-English speaking groups. Interventions can include panel discussions, increased cultural diversity during case-based learning, recruitment and retention programs for cultural minority students, and increased training on identifying and mitigating implicit biases (Burke et al., 2015). Additionally, the creation of identity-affirming environments may help alleviate feelings of poor fit among first year medical students, especially those from cultural minority groups and first-generation medical students. Creating inclusive environments through intergroup exposure can also help underrepresented students become more comfortable with their learning environment and their peers, aiding in the formation of supportive peer networks. The findings of this study suggest that first-generation medical students may also experience a form of minority stress and thus may benefit from structured guidance on navigating the medical school experience, assistance in the early identification of potential mentors, and formal and informal networking opportunities with peers and practitioners. These changes may benefit students from a variety of underrepresented groups by allowing them to form social capital, create a mental and emotional support system, and receive relevant advice on career and service opportunities.

## Limitations and future directions

While this study was conducted to the best of the researchers’ abilities, some limitations must be acknowledged. This study was conducted at a single academic medical center located within the United States over the course of one year. This offered the researchers valuable insights into the experiences of first- through fourth-year medical students at that institution, but those insights may not be transferable to other medical or healthcare student populations. However, the alignment of the results with existing US-based and international research implies that the findings may apply to various medical student populations, though intentional research would be needed to verify this. The majority of the study population were heterosexual White women, which may further limit transferability. However, that is somewhat reflective of the demographic makeup of the institution at which the study was conducted and extra efforts were taken to ensure a variety of diverse voices were included in the study population.

While transcript checks were used, additional logistical constraints prohibited participants from being contacted to provide feedback on the final themes. To address this, future studies could investigate the studied phenomena across multiple years at multiple institutions using different healthcare trainee cohorts. This work may be able to further define the appropriateness of fit of the TMC, its transferability into international and interprofessional contexts, and ways to improve the acquisition and mastery of clinical communication. The voluntary participation of students may have led to selection bias, as students willing to be interviewed about clinical communication likely held strong opinions about it. All participants expressed excitement at the opportunity to discuss communication and communication skills, so it is unlikely that the perspectives of people with low interest in those subjects were interviewed. To access a broader range of participants, further exploration could be done using questionnaire methods to gauge student agreement with definitions of effective clinical communication, personal identities, and their impact on one another. This approach may be able to capture a broader scope of opinions and experiences with an emphasis on those who do not highly value communication as an essential skill.

The themes that were constructed were reflective of the Transactional Model of Communication, which was used as a deductive framework. This could have potentially limited the analytical process by omitting other communication models or utilizing a purely inductive methodology. This study utilized one-time interviews to investigate individual experiences and perceptions surrounding effective clinical communication. These interviews prompted participants to reflect back on their experiences across the last one to four years, which likely limited the researchers’ ability to track how student perceptions and identities changed over time. In addition, additional depth of information may have been lost based on the ability of participants to recall and share specific formative experiences that occurred up to four years ago. To address some of these limitations, the next logical step would be to expand the research questions to investigate shared experiences and perceptions across multiple healthcare professional cohorts using focus groups. An emphasis could be placed on describing the differences between personal, professional, and interprofessional identities across different healthcare disciplines at different stages in their training and/or practice. Regarding the latter point, interprofessional education and collaboration opportunities have gained significant focus recently, with many schools advocating for increased quality and frequency of interprofessional training. The concept of interprofessional identity is a topic of much debate, with several recent reviews identifying incongruent definitions and supporting the need for consistent conceptualization in education and scholarship (Cantaert et al., 2022; Tong et al., 2020). While this study did not investigate the role of interprofessional identity on effective clinical communication, it is likely an area where further exploration is both warranted and necessary. This could further expand the transferability, timeliness, and richness of data across multiple healthcare trainee populations.

This study was limited by the Covid-19 pandemic which impacted students’ training, ability to interface with patients, peers, and instructions, and telehealth proficiency. Further investigation may be warranted into the long-term effects of the Covid-19 pandemic on medical training and the delivery of and access to healthcare, but many studies have been published on these effects in the last several years. Finally, this study had minor challenges with participant recruitment with the third-year population being somewhat underrepresented compared to other cohorts. Nevertheless, the data for each cohort reached saturation and so the researchers do not feel that there was a gap in the results.

## Conclusions

Based on interviews with first- through fourth-year medical students, the researchers were able to construct a preliminary model for establishing effective clinical communication in medical trainee populations based on the Transactional Model of Communication. This study provided new information about the student educational experience at a single US allopathic school, which may offer broader insights about identity concordance and intersectionality in healthcare communication.

Becoming an effective clinician does not only revolve around medical knowledge expertise, but also the ability to communicate and collaborate. Incorporating these changes into medical curricula may contribute to the ongoing paradigm shift that centralizes patient-centered care and effective communication over the simple recitation of factual knowledge (Veazey, 2023). As one student succinctly stated, “Communication is really important … If you’re not a good communicator, you cannot be a good doctor” (M1-2).

## Supplementary Information

Below is the link to the electronic supplementary material.

Supplementary Material 1

Supplementary Material 2

Supplementary Material 3

## Data Availability

Due to the identifiable and sensitive nature of the transcripts, they are not available to view. The codebooks generated during and/or analyzed during the current study are available from the corresponding author on reasonable request.

## Footnotes

1 One student asked that their roommate be present.
