# Supplementary material for: Medical student perceptions of establishing effective clinical communication: a qualitative study
Source: Adv Health Sci Educ Theory Pract. 2025 Aug 14;31(2):653–81. doi: 10.1007/s10459-025-10468-x (PMC13046580; doi:10.1007/s10459-025-10468-x)
Supplement: Supplementary file 1 — Supplementary Material 1 [file 10459_2025_10468_MOESM1_ESM.docx]

**Medical Student Perceptions of Establishing Effective Clinical Communication: A Qualitative Study**

Kathryn Veazey^1^, Andrew Notebaert^2^, Ellen M Robertson^3^

^1^Department of Neurobiology and Anatomy, Drexel University College of Medicine, Wyomissing, PA, USA

ORCID ID: <https://orcid.org/0000-0003-2795-1183>

^2^College of Health and Human Sciences, Northern Illinois University, DeKalb, IL, USA

ORCID ID: [https://orcid.org/0009-0008-1749-9035](https://nam10.safelinks.protection.outlook.com/?url=https%3A%2F%2Forcid.org%2F0009-0008-1749-9035&data=05%7C01%7Ckv452%40drexel.edu%7C1acb7eb55ce744366ab908dbdf108208%7C3664e6fa47bd45a696708c4f080f8ca6%7C0%7C0%7C638349034250904112%7CUnknown%7CTWFpbGZsb3d8eyJWIjoiMC4wLjAwMDAiLCJQIjoiV2luMzIiLCJBTiI6Ik1haWwiLCJXVCI6Mn0%3D%7C3000%7C%7C%7C&sdata=ujrRZjjXEnJlz71OXsWMo%2BOMbBlMCBYHr6m01VU7ACc%3D&reserved=0)

^3^Department of Physician Assistant Studies, Randolph-Macon College, Ashland, VA, USA

ORCID ID: <https://orcid.org/0000-0001-7967-6588>

Correspondence to: Dr. Kathryn Veazey, Department of Neurobiology and Anatomy, Drexel University College of Medicine, 50 Innovation Way, Wyomissing, PA 19610. E-mail: [kv452@drexel.edu](mailto:kv452@drexel.edu)

Submitted for consideration to: *Advances in Health Sciences Education*

## Supplement 1: Semi Structured Interview Script

1. Introduction of Investigator and Research Study
   1. Who I am
      1. Kathryn Veazey, graduate student in Clinical Anatomy PhD program in Department of Neurobiology and Anatomical Sciences
      2. Primary point of contact for this research study
      3. This study is being supervised by my research mentor, Dr. Ellen Robertson
      4. Collecting data on clinical communication skills in medical students for my dissertation project
   2. Explanation of study
      1. What they will be asked to do
         1. Answer a series of questions regarding their individual experiences and how those experiences have influenced how they have gained clinical communication skills
         2. Follow up within 6 months to review interview transcript and confirm that interviewer has accurately depicted the participants’ experience
            1. Option to provide additional elaboration or commentary
      2. Risks and Benefits
         1. Your participation in this study is voluntary and you are free to withdraw at any time. You can also choose to skip questions by saying “pass”.
         2. You will not receive a direct benefit from participating in this study; however, your responses may help us to identify ways to improve future clinical skills training programs which could increase patient satisfaction, improve patient health outcomes, reduce incidence of medical malpractice, and reduce stress and anxiety of healthcare professionals.
         3. Potential risks for participating in this study include emotional discomfort and breach of confidentiality. Should you feel emotional discomfort, you can seek support through the Student Counseling and Wellness Center. To protect confidentiality, only I (Kathryn Veazey) will have access to your name and email address which will be removed from the interview transcript following the 6 month check in. Your participation is confidential. No identifying information will be used. Any published results will be deidentified and reported in aggregate. Pseudonyms will be used for any quotes.
   3. Purpose of study
      1. Identify the factors that influence how medical students believe they gain clinical communication skills
      2. Explore how those factors evolve over the course of the medical school experience
   4. Why they were chosen to participate
   5. Solicit questions from participant regarding study’s design or execution
2. Confirm demographic information
   1. Year in medical school (Class of 202x)
   2. Age
   3. Race or ethnicity
   4. Gender
   5. Sexual orientation
   6. Healthcare provider parents
   7. Married
   8. First-generation medical student
   9. Immigrant or child of immigrants
   10. Religious
3. Open-ended questions
   1. *We are now going to begin the interview process. I am going to start by asking a series of questions about your perceptions of clinical communication skills and your experiences during medical school.*
   2. How would you define or describe clinical communication skills?
      1. Can you provide some examples of good clinical communication skills?
         1. Why are they good examples?
      2. Can you provide some examples of bad clinical communication skills?
         1. Why are they bad examples?
      3. What are some of the consequences of having bad clinical communication skills?
      4. This study’s definition of clinical communication skills is as follows:
         1. *A set of observable behaviors that are demonstrated in clinical or healthcare-related settings to solicit information, establish rapport, and improve patient compliance and long-term health outcomes. These behaviors include taking a patient history, delivering bad news, making empathetic statements, maintaining appropriate silence, and encouraging and answering patient questions. Clinical communication occurs between clinicians, patients, the patient’s family members, and other members of the healthcare team.*
         2. Would you like to make any changes to your definition of clinical communication skills? Why or why not?
   3. How do you feel about being taught clinical communication skills as a part of the medical program?
      1. How do you think your feelings about clinical communication skills training influences how you communicate in clinical settings?
   4. How would you describe effective communication between a doctor and a patient? Can you provide some good and bad examples?
   5. How would you describe effective communication between a doctor and a patient’s family? Can you provide some good and bad examples?
   6. How would you describe effective communication between a doctor and other members of the healthcare team? Can you provide some good and bad examples?
   7. How would you describe your role as a member of the healthcare team?
      1. How do you think your perceptions of effective communication influence how you communicate in clinical settings?
      2. How has your training influenced your perceptions of effective communication?
   8. How does your clinical approach change if you are talking with someone of a different gender, ethnicity, or sexual orientation?
   9. How does your clinical approach change if you are talking to someone who struggles to communicate in English?
   10. How does your clinical approach change if you are talking to someone who you already have an established relationship with versus someone you’re meeting for the first time?
       1. How has your training influenced how you would change your approach in these different scenarios?
          1. Did your training prepare you for these different scenarios?
   11. What do you think a person needs to be a “good” communicator?
   12. What do you think a person needs to be a “good” doctor?
   13. How would you describe yourself as a communicator?
       1. What clinical communication skills do you believe you can improve upon?
       2. How do you think your perceptions of your own abilities as a communicator influence how you communicate in clinical settings?
          1. How has your training influenced those perceptions?
   14. What would you say were the main experiences from the medical program that have changed how you communicate?
       1. In what ways have they influenced how you communicate?
   15. Were there any experiences from the medical program that you feel hindered your communication skills learning? If so, which experiences and how?
   16. *I am now going to ask a series of questions regarding your background, upbringing, and experiences prior to medical school.*
   17. Why did you choose to pursue a career in medicine?
       1. How do you think your reason for pursuing medicine influences how you communicate in clinical settings?
          1. Has that reason changed as a result of your training? If so, how?
   18. How would you describe your personality?
       1. How do you think your personality influences how you communicate in clinical settings?
          1. Has your personality changed as a result of your training? If so, how?
   19. What assumptions do you think other people make about you based on your appearance at the start of a communication encounter (ex. patients, co-workers, supervisors)?
       1. How do you think your awareness of those assumptions influences how you communicate in clinical settings?
          1. Has that awareness changed as a result of your training? If so, how?
   20. Do you consider yourself a non-traditional student?
       1. How do you think your identity as a non-traditional student influences how you communicate in clinical settings?
          1. Has that influence changed as a result of your training? If so, how?
   21. How would you describe your upbringing and childhood?
       - 1. Did you feel encouraged to participate in family discussions or activities?
         2. Were you involved with any large social groups?
       1. How do you think your upbringing influences how you communicate in clinical settings?
          1. Has that influence changed as a result of your training? If so, how?
   22. Did you have any conditions (medical or otherwise) that altered your physical presentation or manner of speaking?
       1. How do you think that condition influences how you communicate in clinical settings?
          1. Has that influence changed as a result of your training? If so, how?
   23. Did you have any conditions (medical or otherwise) that altered your mental health?
       1. How do you think that condition influences how you communicate in clinical settings?
          1. Has that influence changed as a result of your training? If so, how?
   24. How would you describe your identity?
       1. How do you think your identity influences how you communicate in clinical settings?
          1. Has the way that you identify changed as a result of your training? If so, how?
   25. Is there anything else you would like to add about how your individual experience influenced how you learned to communicate?
   26. *Thank you for your time. That concludes the interview questions.*
4. Request approval for follow-up in the next 6 months
   1. Check for clarification and accuracy of transcription
   2. Provide additional elaboration if needed
5. Request additional volunteers
   1. Please pass along the recruitment letter to two other medical students in your year:
      1. 1 student who you believe has similar experiences to you
      2. 1 student who you believe has different experiences from you
