# Supplementary material for: Medical student perceptions of establishing effective clinical communication: a qualitative study
Source: Adv Health Sci Educ Theory Pract. 2025 Aug 14;31(2):653–81. doi: 10.1007/s10459-025-10468-x (PMC13046580; doi:10.1007/s10459-025-10468-x)
Supplement: Supplementary file 3 — Supplementary Material 3 [file 10459_2025_10468_MOESM3_ESM.docx]

**Medical Student Perceptions of Establishing Effective Clinical Communication: A Qualitative Study**

Kathryn Veazey^1^, Andrew Notebaert^2^, Ellen M Robertson^3^

^1^Department of Neurobiology and Anatomy, Drexel University College of Medicine, Wyomissing, PA, USA

ORCID ID: <https://orcid.org/0000-0003-2795-1183>

^2^College of Health and Human Sciences, Northern Illinois University, DeKalb, IL, USA

ORCID ID: [https://orcid.org/0009-0008-1749-9035](https://nam10.safelinks.protection.outlook.com/?url=https%3A%2F%2Forcid.org%2F0009-0008-1749-9035&data=05%7C01%7Ckv452%40drexel.edu%7C1acb7eb55ce744366ab908dbdf108208%7C3664e6fa47bd45a696708c4f080f8ca6%7C0%7C0%7C638349034250904112%7CUnknown%7CTWFpbGZsb3d8eyJWIjoiMC4wLjAwMDAiLCJQIjoiV2luMzIiLCJBTiI6Ik1haWwiLCJXVCI6Mn0%3D%7C3000%7C%7C%7C&sdata=ujrRZjjXEnJlz71OXsWMo%2BOMbBlMCBYHr6m01VU7ACc%3D&reserved=0)

^3^Department of Physician Assistant Studies, Randolph-Macon College, Ashland, VA, USA

ORCID ID: <https://orcid.org/0000-0001-7967-6588>

Correspondence to: Dr. Kathryn Veazey, Department of Neurobiology and Anatomy, Drexel University College of Medicine, 50 Innovation Way, Wyomissing, PA 19610. E-mail: [kv452@drexel.edu](mailto:kv452@drexel.edu)

Submitted for consideration to: *Advances in Health Sciences Education*

**Supplement 3: Reflexivity Statements**

All educator-researchers were based at UMMC at the time that the study was conducted. The first author (KV) identifies as a non-religious queer White ciswoman of Hispanic descent with no known disability. At the time the study was conducted, she was a PhD student and teaching assistant at UMMC. The second author (AJN) identifies as a non-religious straight White cisman with no known disability. He is a health professions educator and directs a doctoral program in the Health Sciences. The third author (EMR) identifies as a religious straight White ciswoman with no known disability. She is a registered and licensed occupational therapist and anatomy educator. The authors acknowledge that their identities may have resulted in biases regarding a variety of demographic characteristics, including gender, sexual orientation, race or ethnicity, religiosity, disability, immigrant experience, and first-generation medical student experience (Veazey, 2023).

All researchers have previous experience conducting mixed methods, quantitative, and qualitative research. This project was KV’s first formalized interview study. To prepare, she received informal training from EMR on conducting and analyzing semi-structured interviews. She also conducted a literature review of proper interview procedures, as well as piloted the interview script prior to data collection. Despite this, there was significant room for improvement in the first three interviews regarding the flow of information, transcription of field notes, and emotional preparation required for conducting interviews surrounding personal identity. As the study progressed, KV became more skilled at redirecting conversations towards the research topic, offering space and verbal affirmations to participants in sensitive moments, and provided student counseling resources when appropriate (Veazey, 2023).

This project was conducted as part of KV’s PhD research, the full results of which have been published (Veazey, 2023). However, this manuscript expands on the previous work by exploring the complexities of interprofessional identity, restructuring the model for effective clinical communication, and concisely summarizing specific recommendations for healthcare educators to improve how students acquire and utilize effective clinical communication. The published form of the manuscript also aimed to increase access to this study’s results to a broader population of healthcare professionals and students.

None of the investigators were medical doctors or had taken the clinical communications curriculum that the students had. As such, they were functionally outsiders to many of the experiences that students discussed. To address this, the first author collected the syllabi and schedules from the relevant coursework that students received, as well as met with some of the clinical education directors before the study’s initiation to clarify her understandings of the curriculum, its objectives, and its assessment methods (Veazey, 2023).

All researchers had prior relationships with participants. Both EMR and AJN served as anatomy faculty for the M1, M2 and M3 cohorts. AJN served as anatomy faculty for the M4 cohort, while EMR served as a teaching assistant for the M4 cohort. KV served as a teaching assistant for the M2 and M3 cohorts and had taken concurrent coursework with some of the M4s during their first year. KV had no prior relationships with the M1s. To minimize coercion, all interviews were conducted entirely by KV after the conclusion of the students’ preclinical anatomy coursework. Following their interview, nearly all participants thanked KV for providing them with a safe space to share their experiences. Many participants also shared that the interview process was the first occasion they had had to critically reflect on some of their experiences and how those experiences impacted their personal and professional growth as clinical communicators. The study population was surprisingly diverse compared to the researcher’s initial expectations. Most participants identified as first-generation medical students and while many identified as White cisgender heterosexual persons, there were a substantial number of participants who identified as immigrants, people of color, or LGBTQIA+. Many students also shared their experiences with illness or disability, which the investigators had not included in their demographic data collection (Veazey, 2023).

The researchers used the COREQ checklist, developed by Tong et al. to provide explicit, comprehensive reporting of qualitative studies (Tong et al., 2007). However, this checklist has faced recent criticism based on its origins and stated applications (Braun & Clarke, 2024). Braun and Clarke posit that the use of the COREQ checklist is inappropriate to non-positivist frameworks and may present unique challenges for novice qualitative researchers. Given that this was KV’s first large-scale qualitative project and the use of an interpretive framework, this bears discussion. The authors acknowledge that adherence to the COREQ checklist does not ensure rigor, trustworthiness, or quality (Braun & Clarke, 2024). Instead, they feel that it serves as a stepping stone for researchers (especially novices) to use when designing a qualitative study to consider various factors they may have overlooked (such as the qualifications of the research team, the training received by the lead interviewer, and whether previous relationships were established with participants). As with the checklists referenced in the main manuscript, we feel that using a specific scale-based rubric can provide significant value, providing it is used critically and reflectively. The authors implemented multiple additional methods to ensure qualitative rigor, which were outlined in Table 1 of the main manuscript.

Although the authors ultimately developed a theoretical model for effective clinical communication, as well as engaged in theoretical sampling, followed by open and axial coding, it would not be appropriate to state that the authors utilized a grounded theory approach. They had determined prior to the project’s start that a grounded theory approach was inappropriate as there were pre-existing models for effective communication (i.e., the Transaction Model of Communication). Although grounded theory can be applied to existing incomplete models, the use of a deductive coding schema during the initial analysis phase would be inappropriate to that methodology (Delve, 2021). They also did not engage in selective coding, but instead engaged in thematic construction.

The initial analysis plan had been to conduct a first-pass deductive coding rooted in the Transaction Model of Communication, followed by a second-pass inductive coding to identify gaps. However, the deductive codebook was identified to be insufficient early in the transcription process, resulting in only a subset of M3 and M4 transcripts receiving deductive coding. Clean copies of all M3 and M4 transcripts then were coded using inductive methods, which resulted in over 1000 discrete codes. These codes were then consolidated into categories, which were modified iteratively and applied to all transcripts. Lastly, the researchers had initially begun the project by focusing on clinical communication skills; however, participant experiences quickly illuminated that effective clinical communication did not occur as a skill-based practice, but instead resulted from the multifaceted interactions between the communicators involved (Veazey, 2023). This was discussed more thoroughly in the discussion.

**References**

Braun, V., & Clarke, V. (2024). How do you solve a problem like COREQ? A critique of Tong et al.’s (2007) Consolidated Criteria for Reporting Qualitative Research. *Methods in Psychology*, *11*, 100155. <https://doi.org/https://doi.org/10.1016/j.metip.2024.100155>

Delve, H., L., & Limpaecher, A. (2021). The Practical Guide to Grounded Theory. *Practical Guide to Grounded Theory Research*. <https://delvetool.com/groundedtheory>

Tong, A., Sainsbury, P., & Craig, J. (2007). Consolidated criteria for reporting qualitative research (COREQ): a 32-item checklist for interviews and focus groups. *International Journal for Quality in Health Care*, *19*(6), 349-357. <https://doi.org/10.1093/intqhc/mzm042>

Veazey, K. (2023). *Clinical Communication Skills in Medical Students: Influences, Attitudes, and Evolution*. ProQuest Dissertations & Theses.
